# The role of residential mobility in reproducing socioeconomic stratification during the transition to adulthood

**DOI:** 10.4054/DemRes.2018.38.7

**Published:** 2018-01-12

**Authors:** Anne Clark

**Affiliations:** 1 University of Michigan, Department of Sociology and Population Studies Center, Institute for Social Research, Michigan, USA. accla@umich.edu.

## Abstract

**OBJECTIVE:**

This study assesses whether frequency of residential mobility plays a role in the reproduction of socioeconomic inequality during the transition to adulthood based on two criteria: (1) selection – is there socioeconomic sorting into residential trajectories? – and (2) lack of moderation – is this sorting irreducible to other life events that prompt moves (e.g., changes in employment status)?

**METHODS:**

I use two and a half years of monthly address data from the Relationship Dynamics and Social Life data set, a sample of 18- and 19-year-old young women in a Michigan county. As an improvement upon previous measures of residential mobility, I use group-based trajectory analysis to categorize young women into residential trajectory groups. I then conduct a series of nested logistic regressions to predict membership in residential trajectory groups and a decomposition analysis to determine whether rapid movers are exposed to more life events (e.g., entering/exiting employment) or are simply more sensitive to moving in the face of life events compared to gradual movers.

**RESULTS:**

Rapid moving is associated with low socioeconomic status. Rapid movers experience similar family formation, employment, and academic changes as gradual movers but are more likely to move when faced with these life events.

**CONCLUSIONS:**

High residential mobility is a phenomenon among early home-leavers as part of an accelerated and underfunded transition to adulthood rather than a reflection of the upward socioeconomic mobility of college students.

**CONTRIBUTION:**

High residential mobility is not simply a neutral or normative aspect of the transition to adulthood but rather part of the process of reproducing socioeconomic stratification.

## 1. Introduction

Most studies of residential mobility during the transition to adulthood in the United States focus on either home-leaving (and its counterpart, ‘boomeranging’ back to the family home) or locational attainment. The former line of inquiry emphasizes the implications of the timing of residential independence for socioeconomic attainment and an overall healthy and successful progression through life course stages (Aassve, Cottini, and Vitali 2013; Aquilino 1991; Buck and Scott 1993; Goldscheider and Goldscheider 1998; Kahn, Goldscheider, and García-Manglano 2013; Lei and South 2016; Leopold 2012; Sandberg-Thoma, Snyder, and Jang 2015; White and Lacy 1997). The latter, a more recent development, investigates residential mobility outside of the parental home as an important contributor to the reproduction of socioeconomic inequality during the transition to adulthood (Sharkey 2012; Swisher, Kuhl, and Chavez 2013). Both literatures tend to emphasize individual moves or transitions (e.g., between two neighborhoods) and whether those moves are advantageous or disadvantageous to young adults’ long-term socioeconomic attainment.

What remains unclear is the role of frequency of residential mobility in reproducing socioeconomic inequality. Frequent residential mobility during the transition to adulthood is often assumed in the life course literature to be a normative consequence of educational, career, and romantic exploration (Arnett 2000; Jang and Snyder 2015; Michielin and Mulder 2008; White and Lacy 1997). In contrast, research on housing instability has found that frequent residential mobility is a phenomenon among individuals with low socioeconomic status (Astone and McLanahan 1994; Burgard, Seefeldt, and Zelner 2012) that is associated with negative outcomes in the realms of education, employment, food security, and health (Boynton-Jarrett, Hair, and Zuckerman 2013; Desmond 2016; Desmond and Gershenson 2016; Desmond and Kimbro 2015; Fowler, Henry, and Marcal 2015). These two perspectives yield very different assumptions about who moves during the transition to adulthood and how these moves are associated with trajectories of status attainment.

Therefore, the present study asks: Does high residential mobility play a role in the reproduction of socioeconomic stratification during the transition to adulthood? I assess this question using two criteria: selection and lack of moderation. Young adults with low socioeconomic status (SES) must be sorted into trajectories of rapid residential mobility (selection) and differences across residential trajectories cannot simply be a symptom of differences in educational, occupational, and romantic careers (lack of moderation).

I use two and a half years of monthly move data for a random, population-based sample of 868 18- and 19-year-old women in a Michigan county from the Relationship Dynamics and Social Life (RDSL) Study. RDSL represents an improvement over previous longitudinal data sets in capturing residential mobility in that monthly data collection promotes greater accuracy among my key subpopulation of interest: those experiencing housing instability. The high level of detail in the residential data allows me to combine number and timing of moves in the form of residential trajectories. Trajectories are more nuanced than traditional measures of high residential mobility, such as a threshold number of moves per time period (e.g., three moves per year or three moves per three years) (Cutuli et al. 2013; Pavao et al. 2007). Given that the transition to adulthood is the period of the life course with the highest rates of residential mobility (Arnett 2000), measures that incorporate more detail are crucial in order to better distinguish between normative and potentially disadvantageous moving behaviors. The high level of detail in RDSL, leveraged using trajectory methods, yields new insight into the relationship between residential mobility and stratification during the transition to adulthood and provides evidence for considering residential mobility as an independent form of inequality that is irreducible to other life events.

## 2. Background

### 2.1 Selection

The transition to adulthood is a distinct life course stage when frequent residential mobility is normative (Arnett 2000). Young adults, typically defined as aged 18–25, experience frequent change as they explore occupations, romantic interests, and worldviews. Entrances into and exits from schools, jobs, and cohabiting relationships often are accompanied by moves. As a result, mobility peaks during young adulthood and declines thereafter: 33% of 18–25-year-olds have moved at least once in the past year, compared to 17% of 30–44-year-olds and 9% of 45–64-year-olds (author calculations using data from the 2008–2010 American Community Survey).

Perceptions of how residential mobility varies by SES during the transition to adulthood differ in the transition to adulthood literature compared to the housing literature. Some in the transition to adulthood tradition may assume that higher residential mobility is a function of college attendance (Lei and South 2016), especially given the expansion of access to postsecondary education over the past 20 years (National Center for Education Statistics 2015). Moves may therefore be associated with upwardly mobile or neutral life events – such as entering school, summer break, graduation, and career acquisition – among individuals with high SES. However, only 43% of young adults enroll in a postsecondary program (Snyder and Dillow 2015), and only 52% of those students move out of the parental home to attend college (Bozick 2007). This leaves a significant number of moves during young adulthood unaccounted for.

In contrast, researchers in the housing instability tradition assume that most high residential mobility is a form of housing instability (Cutuli et al. 2013; Pavao et al. 2007). Housing instability can be conceptualized broadly as a period of financial distress accompanied by tenuous housing and/or residential mobility. If unchecked, housing instability can eventually lead to homelessness (Desmond 2016). In this framework, frequent moving may be a phenomenon among low-SES young adults who lack the resources to maintain their housing when faced with job loss, relationship instability, or start-and-stop schooling (Curtis et al. 2013). The ability of young adults to support an independent household may be dependent on multiple interrelated factors, including their own financial resources, the availability of family support, and household size (i.e., having to support additional people in the form of a partner and/or children) (Desmond and Gershenson 2017; Desmond, Gershenson, and Kiviat 2015).

### 2.2 Lack of moderation

If rapid movers have low SES, they should experience more life events reflective of educational, occupational, and romantic instability. Low-SES young adults are less likely to pursue postsecondary education (Bozick and DeLuca 2011). However, if they do, they are more likely to delay enrollment (Bozick and DeLuca 2005) and experience interrupted enrollment (Goldrick-Rab 2006). While college students and college graduates are exploring potential career paths, their less-advantaged peers experience “fragmented transitions into work” (Shanahan 2000). High school graduates face either prolonged periods of unemployment or a ‘career’ consisting of a series of low-paid, high-turnover jobs (Rosenbaum 2001). Low-income young adults are also more likely to experience cycles of relationship dissolution and reconciliation (Halpern-Meekin et al. 2013). Low-SES young women are much more likely to become single or cohabiting mothers during the transition to adulthood than their high-SES counterparts, who are more likely to delay family formation to complete their postsecondary education and establish a career (Amato et al. 2008). It is possible, then, that these stratified experiences yield more frequent moves for young adults with low SES (Warner and Sharp 2016), perhaps in ways that reify or exacerbate inequality in other areas of life.

However, differences in residential trajectories may not simply be a symptom of differences in educational, occupational, and romantic careers. If moves are associated with life events there are two factors that can explain greater residential mobility in some groups of movers but not others: differences in exposure and differences in sensitivity. Either more frequent movers are exposed to more life events, necessitating more frequent moving, or they are more sensitive to these events, resulting in a greater likelihood of moving when they happen, whether voluntarily or involuntarily. For example, more frequent movers may move from job to job or experience intermittent school enrollment with periods of working in-between, necessitating more moves. Alternately, even when faced with the same life event, low-SES individuals are more likely to experience a move, whether involuntarily (e.g., because they have no financial buffer to pay rent during unemployment) or voluntarily (e.g., because an increase in income facilitates a move out of a temporary and undesirable living situation). Most likely, both greater exposure and greater sensitivity explain differences in moving behavior. This is consistent with the assumptions of the housing instability literature, according to which housing instability is the result of both financial shocks (e.g., job loss, medical emergency) and the absence of resources to weather those shocks (Curtis et al. 2013).

## 3. Data and methods

### 3.1 Relationship Dynamics and Social Life Study

I use data from the Relationship Dynamics and Social Life (RDSL) Study, a longitudinal survey based on a random, population-based sample of 1,003 18- and 19-year-old women in a Michigan county. Respondents were drawn from state driver’s license and ID card records. After a face-to-face baseline interview conducted between March 2008 and July 2009, respondents completed weekly interviews for two and a half years via a secure website or phone. Of the women located, 93% participated in the baseline interview, and more than 72% of women interviewed at baseline remained in the study for at least 18 months. This analysis uses a sample of 868 respondents who remained in the study for at least two months, the minimum amount of time necessary to encounter the residential change question at least twice.

RDSL provides unique insight into the residential trajectories of young women for multiple reasons. First, monthly measures of mobility for a large, population-representative sample yield the most accurate picture of residential mobility patterns to date. Second, although RDSL data is drawn from a tight cohort in only one county, this ensures that the respondents face the same housing and employment markets, educational opportunities, and any other time-varying and geographic factors that influence residential mobility. Third, analyzing young women separately from young men eliminates the need to disentangle gendered family formation (Meier and Allen 2008; Mollborn 2007), home-leaving (Avery, Goldscheider, and Speare 1992; Buck and Scott 1993), and rental processes (Desmond 2012) that lead to gendered experiences of housing instability and homelessness (Desmond 2012; US Department of Housing and Urban Development 2014). And finally, while RDSL does not capture all of the transition to adulthood, which traditionally comprises ages 18–25, it does capture the period with the most heterogeneity, mobility, and participation in activity typical for that stage of the life course (Arnett 2000). The age range of RDSL respondents enables me to capture in great detail the residential trajectories of those who leave home early and struggle in establishing stable households.

RDSL samples are from a narrow geographic area. However, this level of detailed mobility data, reported in monthly intervals, is not available in national data sets. Although the National Survey of Families and Households – which is frequently used to investigate home-leaving and returns to the parental home (Aquilino 1991; Goldscheider and Goldscheider 1998; De Marco and Berzin 2008; Tang 1997; White and Lacy 1997) – includes an event history calendar, respondents are asked to recall multiple years of housing history at a time. This traditional panel structure likely yields an undercount of moves in my key subpopulation of interest, rapid movers, especially if they go through periods of couch surfing or other forms of housing instability where exact dates and number of residences may be difficult to recall years later. Given that young adulthood is marked by a great deal of change in multiple areas of life, with significant consequences for future status attainment, more intensive data collection is necessary to accurately capture residential mobility and its interrelationships with romantic, employment, and academic changes.

Although RDSL’s sample generalizes to a single county in Michigan, the respondents are roughly comparable to the US population of 18- and 19-year-old women at the time of data collection, as measured using the 2008–2010 American Community Survey (ACS) 3-year Estimates (calculations available from the author upon request). Less than half of both samples are employed, approximately one-third move over the course of the year, over two-thirds still live with family, and hardly any are married. Since RDSL respondents had to already be 18 when they were sampled, by the time they were interviewed 18-year-olds tended to be older within their 18th year of age (i.e., exact age within 18-year-olds was skewed older). When both the RDSL and ACS samples are limited to 19-year-olds, RDSL respondents have a uniform exact age distribution (age in days unavailable in ACS PUMS) and comparable enrollment in both high school (8% vs. 7% respectively) and postsecondary school (63% vs. 60%) to ACS respondents.

The main difference between the RDSL sample and young women nationally is racial and ethnic composition. Twice as many RDSL respondents are black or African American (34% vs. 17%) and less than half as many are Hispanic (8% vs. 19%). As such, the following analyses are not representative of the residential trajectories of young Latinas nationally.

### 3.2 Measures

#### 3.2.1 Residential mobility

Residential mobility is measured based on addresses provided as contact information, which respondents were asked to update monthly. Addresses are geocoded. Any change in geocode is captured in a time-varying binary move variable. This binary variable is used to construct a variable representing cumulative number of moves outside of the family home: Respondents start with a value of zero or one (for respondents who had already moved out of the family home by baseline, 24%), with one added at each interview reporting a move. Attributing a move at baseline for early home-leavers reduces the impact of unobserved heterogeneity based on left-censoring of home-leaving trajectories, although I cannot deduce the number of moves outside of the family home that occurred before baseline. This cumulative variable assumes that respondents do not return to the family home. Supplemental analyses after the main results will address boomeranging, or returns to the family home after the initial home-leaving move. The cumulative move variable is used to identify groups of respondents with similar residential trajectories using group-based trajectory analysis, as discussed further below. This categorical variable is the measure I ultimately use in my analyses to identify sorting into residential trajectory groups and differences in exposure as opposed to sensitivity to life events across groups.

The 868 respondents in this analysis experienced a total of 1,038 moves during the study. Over the course of the study 43% of respondents never moved, 26% moved once, 15% moved twice, 8% moved three times, and 8% moved four or more times (with a maximum of eight moves).

Each move is coded for context. Specifically, the moves are coded for relationship, employment, and academic changes that occurred concurrently with the move. Relationship changes were measured weekly, residential mobility was measured monthly, and academic and employment changes were measured quarterly. The data does not include when the events occurred in relation to each other.

A single move could be associated with multiple events in other life domains (see Table A-1 in the Appendix for details). Relationship events – entering cohabitation, exiting cohabitation, intimate partner violence, and pregnancy or giving birth – are not mutually exclusive. For example, a woman could both enter cohabitation and become pregnant. In contrast, employment changes – (1) entering a job or changing part-time to full-time and (2) leaving a job or changing full-time to part-time – are mutually exclusive. Academic events include starting school, summer break, transferring to a school granting a higher degree (e.g., from a two-year college to a four-year college), transferring to a school granting a lower degree (e.g., from a four-year college to a two-year college), graduating school, and quitting school. These events are mutually exclusive. There were 14% of moves that could not be linked with a relationship, employment, or academic event.

#### 3.2.2 Baseline characteristics

Childhood and baseline transition to adulthood characteristics predict residential trajectory group membership during the study. These predictors are organized into five groups: childhood background and race, family support, financial resources, education and employment, and family formation.

Childhood background and race are captured by four dichotomous variables, coded to one for each of the following conditions: The respondent identifies as black or African American, the respondent’s mother’s educational attainment is less than a high school diploma or equivalent, the respondent did not grow up with two biological parents or a biological parent and a step-parent, and the respondent’s family ever received public assistance during the respondent’s childhood.

Family support is measured by whether the respondent received financial support from her family at baseline and by whether the respondent was very or extremely close to her mother or father during childhood.

The respondent’s financial resources at baseline are captured by two measures. The first is a binary variable indicating whether the respondent had just enough or not enough money for monthly expenses (as opposed to having some money left over at the end of the month). The second is a binary variable indicating whether the respondent was a recipient of some form of public assistance.

The respondent’s employment at baseline is represented by a dichotomous variable indicating whether the respondent was employed at all. The respondent’s educational enrollment at baseline is captured by a categorical variable with three categories: not enrolled (the reference category), enrolled in high school, and enrolled in postsecondary school.

The respondent’s family formation behavior at baseline is measured by whether the respondent was married, engaged, or cohabiting and whether she had ever experienced a live birth.

### 3.3 Group-based trajectory analysis

Housing instability is often operationalized as a dichotomous variable using a threshold number of moves over a given time period because most measures of housing instability are retrospective over many years. Conceptually, however, housing instability is better reflected by differences in rate of moves across members of a population. For example, some individuals with a high number of moves may move so rapidly that the constant struggle to maintain stable housing is consuming all of their time and resources, while other individuals with a high number of moves may relocate at regular intervals consistent with normative institutional constraints, such as academic years.

Therefore, I construct a categorical measure of residential mobility based on rate of moving via slopes of trajectories. This measurement strategy is made possible by the detailed monthly move data available in RDSL. Trajectories allow moves to be nested within women and contextualized with regards to when they occur in women’s lives and when they occur in relation to each other, consistent with a life course perspective. Specifically, using age-graded trajectories distinguishes between leaving home and subsequently moving at different ages, which is crucial given that even a one- or two-year difference in timing during the transition to adulthood can be substantively meaningful (e.g., the difference between leaving the parental home after high school or after completing an associate’s degree).

I use group-based trajectory analysis (GBTA) as opposed to an alternative such as growth curve modeling (GCM) because a method that yields a single population average trajectory fundamentally fails to distinguish between movers and nonmovers. Furthermore, housing stable movers and housing unstable movers likely experience different trajectories. GBTA assumes that latent statistical processes generate distinct groups with respect to some variable that changes over time or with age (Nagin 2005; Nagin and Odgers 2010; Wagmiller, Jr. et al. 2006). In other words, rather than assuming that the population varies normally around some mean population trajectory (as with GCM), GBTA assumes that a latent variable divides the population into subpopulations, each with its own mean trajectory. Because the variable dividing the population into distinct patterns of behavior is unobserved, group membership must be inferred from the mix of trajectories based on the trajectories’ shape. GBTA can simultaneously estimate the predicted trajectory of each group, the percent of the sampled population in each group, and a respondent’s probability of group membership (i.e., posterior probabilities). I use these posterior probabilities to sort respondents into trajectory groups to generate further descriptive statistics, run a series of nested models predicting group membership, and conduct a decomposition analysis, described below.

The traditional GBTA model assumes that attrition is independent of trajectory group membership. However, nonrandom attrition may impact estimates of trajectory group size, particularly when trajectories are initially not well separated and become more distinct over time, as is the case here. Haviland, Jones, and Nagin (2011) developed an extension of GBTA for correcting estimates of trajectory group size for nonrandom attrition, which I incorporated as a sensitivity analysis using the traj command in Stata. I ran the final model testing for constant attrition and attrition as a function of previous residential mobility. Estimates for group size were stable. Therefore, I present the final model without adjusting for attrition.

### 3.4 Decomposition

Decomposition is closely related to standardization (Das Gupta 1993). Both techniques disentangle differences in subpopulation composition (e.g., age structure) from differences in subpopulation-specific conditional probabilities (e.g., age-specific death rates) which combine to yield differences in overall rates (e.g., crude death rate) across populations. However, while standardization holds composition constant to examine differences in conditional probabilities, decomposition determines the percent of the difference in overall rates attributable to differences in composition and the percent attributable to differences in conditional probabilities.

In this case, differences across mover groups in rates of experiencing move types are attributable to both compositional differences in experiencing a given life event and differences in conditional probabilities of moving given the life event. More substantively, compositional differences can be considered differences in exposure to life events and differences in conditional probability can be considered differences in sensitivity to moving in response to life events. For example, if some groups are more likely to move while entering school, this can be a result of their greater propensity to enter school, their higher probability of moving when they enter school, or both. Broadly, the decomposition method used here can be summarized as follows (Preston, Heuveline, and Guillot 2001):

Differenceinrateofmovetype=differenceinexposuretolifeevent(weightedbyaveragesensitivitytolifeevent)+differenceinsensitivitytolifeevent(weightedbyaverageexposuretolifeevent).

The decomposition will show whether rapid movers move more frequently than other movers because they are exposed to more life events that prompt moves, because they are simply more sensitive to moving when faced with a life event, or both. I bootstrap standard errors to test whether exposure effects and sensitivity effects are significant (Wang et al. 2000).

## 4. Results

The final GBTA model contains three linear residential trajectory groups. These groups comprise a categorical measure of residential mobility used in the following sections to identify socioeconomic selection into trajectory groups and differences in exposure and sensitivity to life events across trajectory groups. The Bayesian information criterion (BIC) improved with the addition of further trajectory groups up to a total of five (the BIC is −13,325 for three groups and −12,825 for five groups). However, beginning with the four-group model, estimates of group size were highly sensitive to the addition of covariates when multinomial logits predicting group membership were estimated simultaneously with the trajectories (e.g., the estimated size of the smallest group in the four-group model ranged from 5% to 13% of the population). This suggests that residential trajectories across groups were no longer distinct, such that baseline characteristics significantly influenced trajectory group membership for individuals whose residential trajectories resembled the average of two adjacent groups. This left group membership highly dependent on the selection of covariates. The final model uses three groups so that residential trajectory groups are empirically distinct.

**Figure 1:**
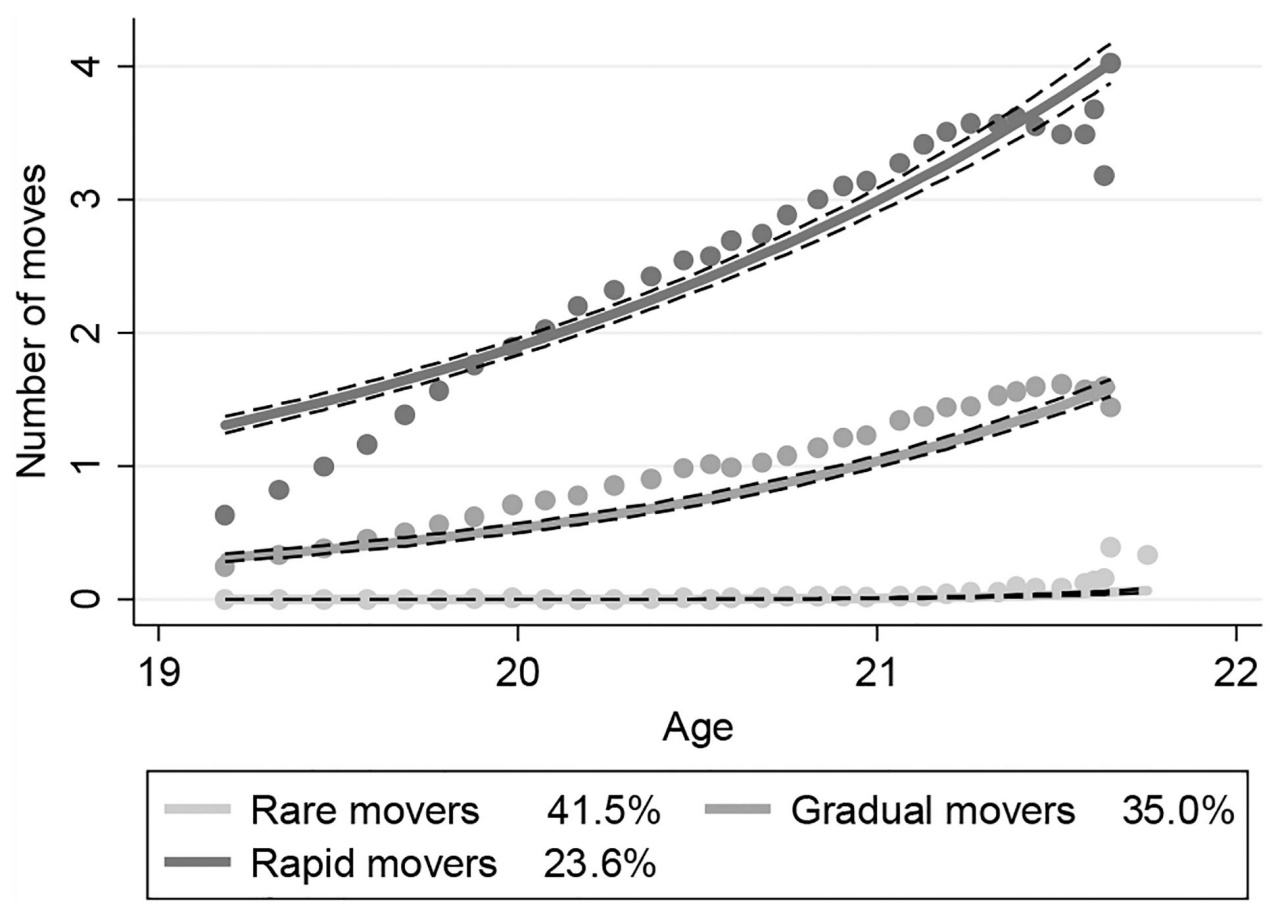
Estimated residential trajectory groups in RDSL, by estimated percent of population Notes: The solid lines represent estimated trajectories. The dashed black lines represent the 95% pointwise confidence intervals on the estimated trajectories. The dots represent observed group means for each trajectory.

**Figure 2:**
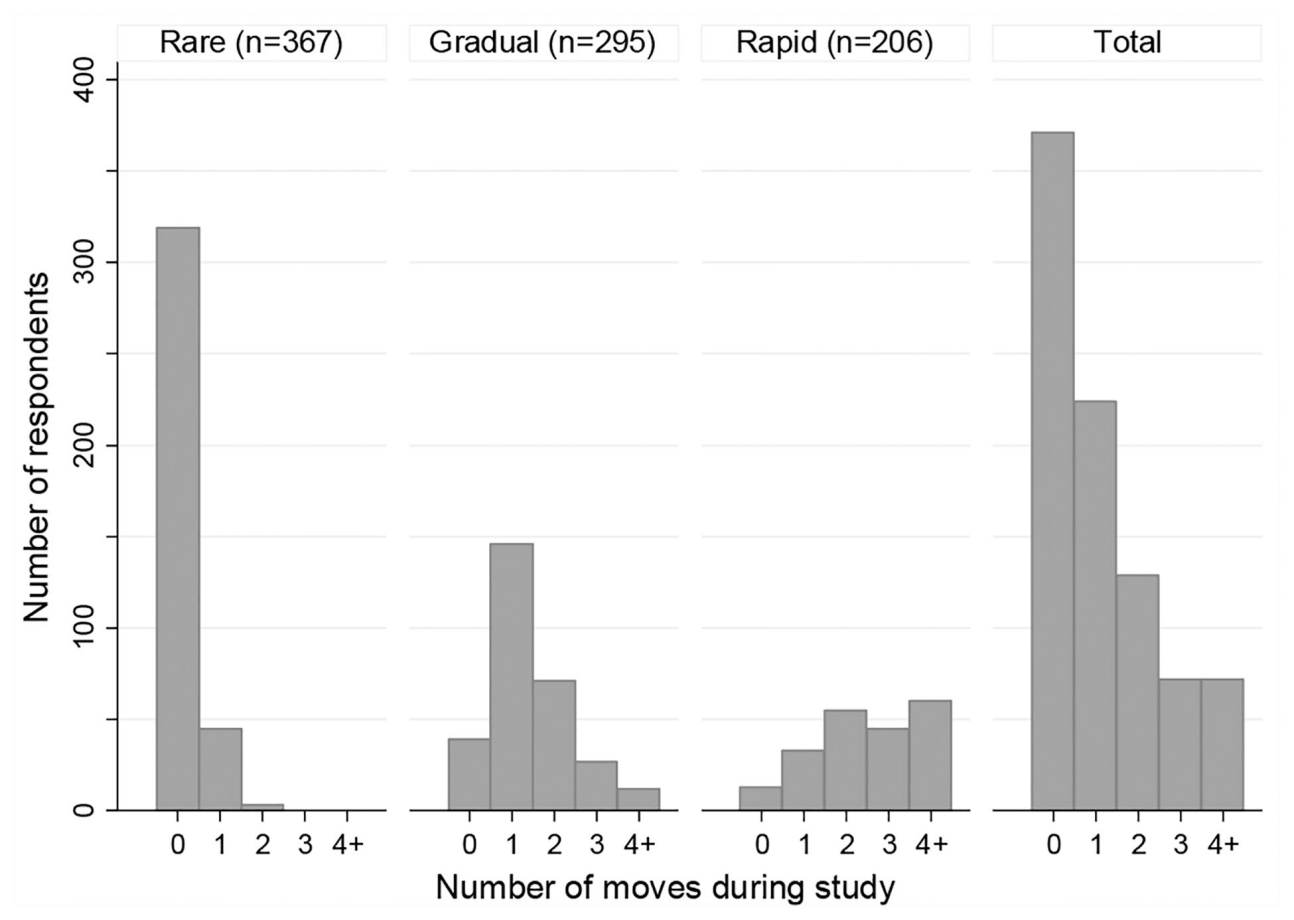
Number of moves during study, by residential trajectory group

Figure 1 displays the results of the GBTA model identifying three distinct residential trajectory groups in this population of young women: rare movers, gradual movers, and rapid movers. The figure depicts estimated trajectories for each trajectory group, the 95% pointwise confidence intervals on the estimated trajectories, and the observed group means for each trajectory. The legend includes the estimated percent of the population in each group. These population estimates are generated by the GBTA model. In contrast, Figure 2 presents sample means calculated by assigning each RDSL respondent to a trajectory group using posterior probabilities generated by the GBTA model. Figure 2 shows the distribution of moves occurring during the study within each trajectory group. Note that women who had already left the family home by baseline (24% of the sample) are categorized as movers due to their early home-leaving, even if they do not move during the observation period.

The largest residential trajectory group (estimated 42% of the population) is characterized by no residential change. Women in this trajectory group will henceforth be called rare movers. All rare movers were living with family at baseline (descriptive statistics for living with family at baseline available upon request). Most (87% of rare movers) do not move over the course of the study, and a small fraction (12%) move once toward the end of the study out of the family home (an additional 1% move twice toward the end of the study; descriptive statistics for timing of moves available upon request). The rare movers have a very stable residential trajectory characterized by residence in the parental home for most if not all of the study period.

The second largest residential trajectory group (35% of the population) is characterized by gradual residential change. Women in this trajectory group will henceforth be called gradual movers. Most gradual movers lived in the family home at baseline (76% of gradual movers) and move once (49%) or twice (24%) over two and a half years, with moves spaced about one year apart. This level of mobility is consistent with young women gradually gaining independence and/or coordinating their housing with the academic calendar.

The final residential trajectory group (24% of the population) is characterized by rapid residential change. Women in this trajectory group will henceforth be called rapid movers. Most rapid movers had moved out of the family home by baseline (65% of rapid movers) and experience moves in rapid succession, with around half (51%) moving three or more times over the course of the study. This mobility pattern is potentially unstable, contingent on other characteristics of the respondents and their moves.

### 4.1 Selection

Table 1 presents the results of two series of nested logistic regressions of baseline characteristics that predict membership in residential trajectory groups. The first series (Models 1–5) predicts whether respondents were movers (i.e., rapid or gradual movers vs. rare movers). The second series (Models 6–10) limits the sample to movers and predicts whether respondents were rapid movers (vs. gradual movers). To shed light on mediated (indirect) effects between low socioeconomic status and patterns of residential mobility, Models 1 and 6 begin with indicators of childhood background and race, which I interpret as exogenous. In the following models, I introduce blocks of variables corresponding to different dimensions of the transition to adulthood: family support, financial resources, education and employment, and family formation.

In the full sample, growing up in a household that received public assistance or was not headed by two parents is significantly associated with residential mobility (Model 1). The effect of childhood poverty is fully mediated by financial resources available to the respondent at baseline: Movers are less likely to receive financial support from family (Model 2) and more likely to have difficulty paying for their expenses and to receive public assistance (Model 3). The effect of growing up without two parents is partially but not fully mediated by these indicators of financial resources. High school enrollment predicts membership in the rare-mover group (Model 4) and being in a serious relationship (married, engaged, or cohabiting) is associated with residential mobility (Model 5). However, neither of these variables significantly mediates the effects of childhood background. Notably, enrollment in postsecondary school does not significantly predict residential mobility (Model 4).

Among movers, growing up in a household that received public assistance is associated with rapid moving (Model 6). The effect of childhood poverty is fully mediated by financial resources: Rapid moving is associated with lack of financial support from family (Model 7) and public assistance receipt at baseline (Model 8). Once again, enrollment in postsecondary school does not significantly predict residential trajectory group membership (Model 9). However, the effect of all SES indicators is fully mediated through relationship status (Model 10). Being married, engaged, or cohabiting at baseline increases the odds of being in the rapid group (as opposed to the gradual group) by 90% (e^64^=1.90).

Overall, this paints a picture of residential mobility that is much more consistent with the struggles of low-SES respondents to support themselves, especially if they have expanded their households, rather than with the neutral or upwardly mobile moves of four-year college students.

Finally, identifying as black or African American is significantly associated with less residential mobility in both sets of models, although the effect of race in the full sample is mediated by relationship status (Model 5). This is consistent with prior research showing that African Americans tend to leave home later (Lei and South 2016; De Marco and Berzin 2008; Tang 1997) and are less mobile than their Caucasian counterparts despite experiencing greater economic pressures to move, such as high individual- and neighborhood-level unemployment rates (Spilimbergo and Ubeda 2004).

### 4.2 Lack of moderation

**Figure 3:**
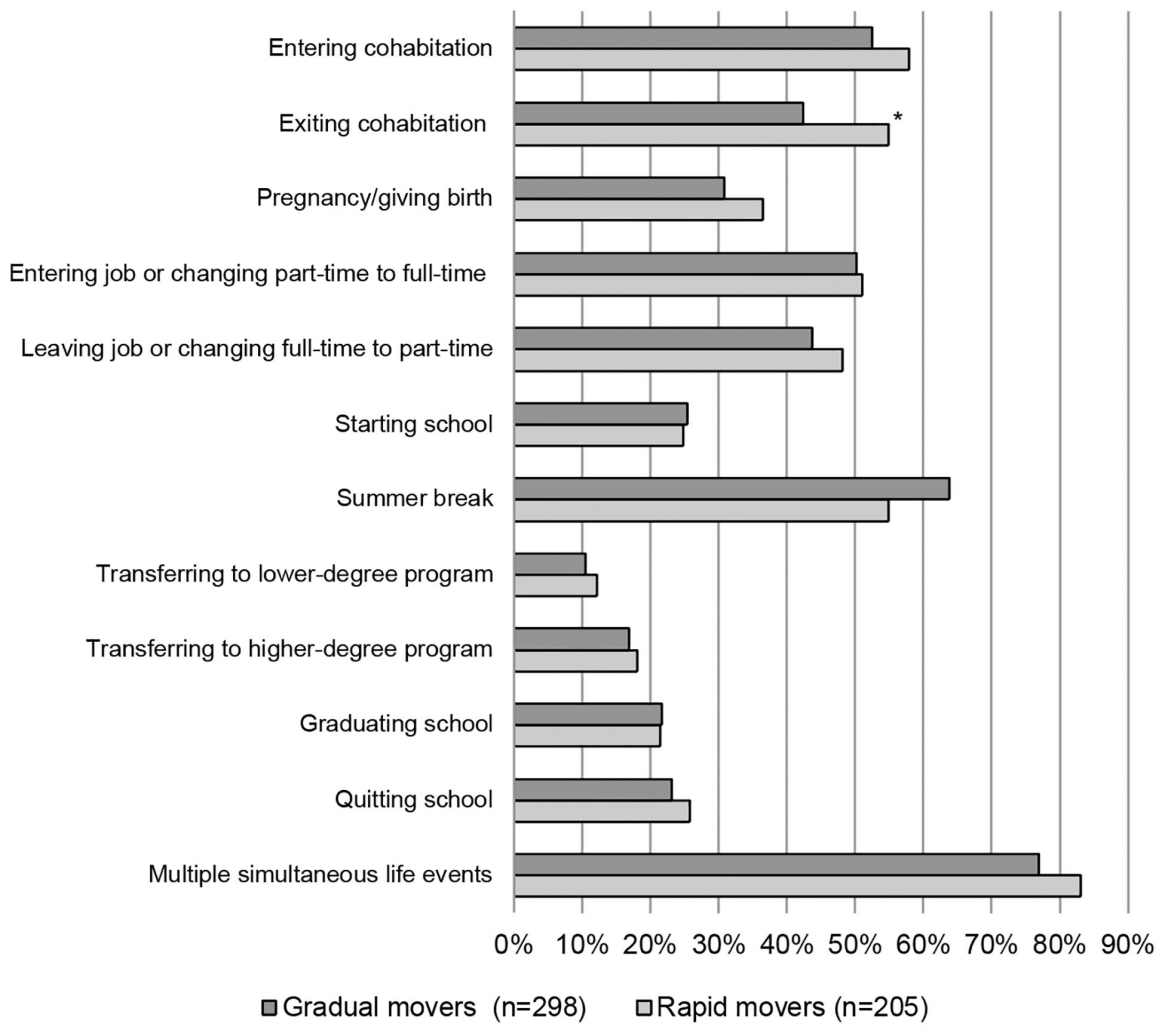
Percent of respondents ever experiencing life event during study (n = 868) *Note*: Chi-square tests identify significant differences across residential trajectory groups. † p < .10. * p < .05. ** p < .01.

Figure 3 presents the percent of gradual and rapid movers who ever experience a variety of relationship, employment, and academic changes and significant between-group differences in exposure using chi-square tests. Rapid movers are significantly more likely to exit cohabitation. Otherwise, rapid movers and gradual movers have very similar relationship, employment, and academic experiences. I also conducted sensitivity analyses (not shown) testing for differences in exposure using number of events instead of ever experiencing an event. Results were substantively the same, the only difference being that the difference in exiting cohabitation was only marginally significant (p < .10).

**Figure 4:**
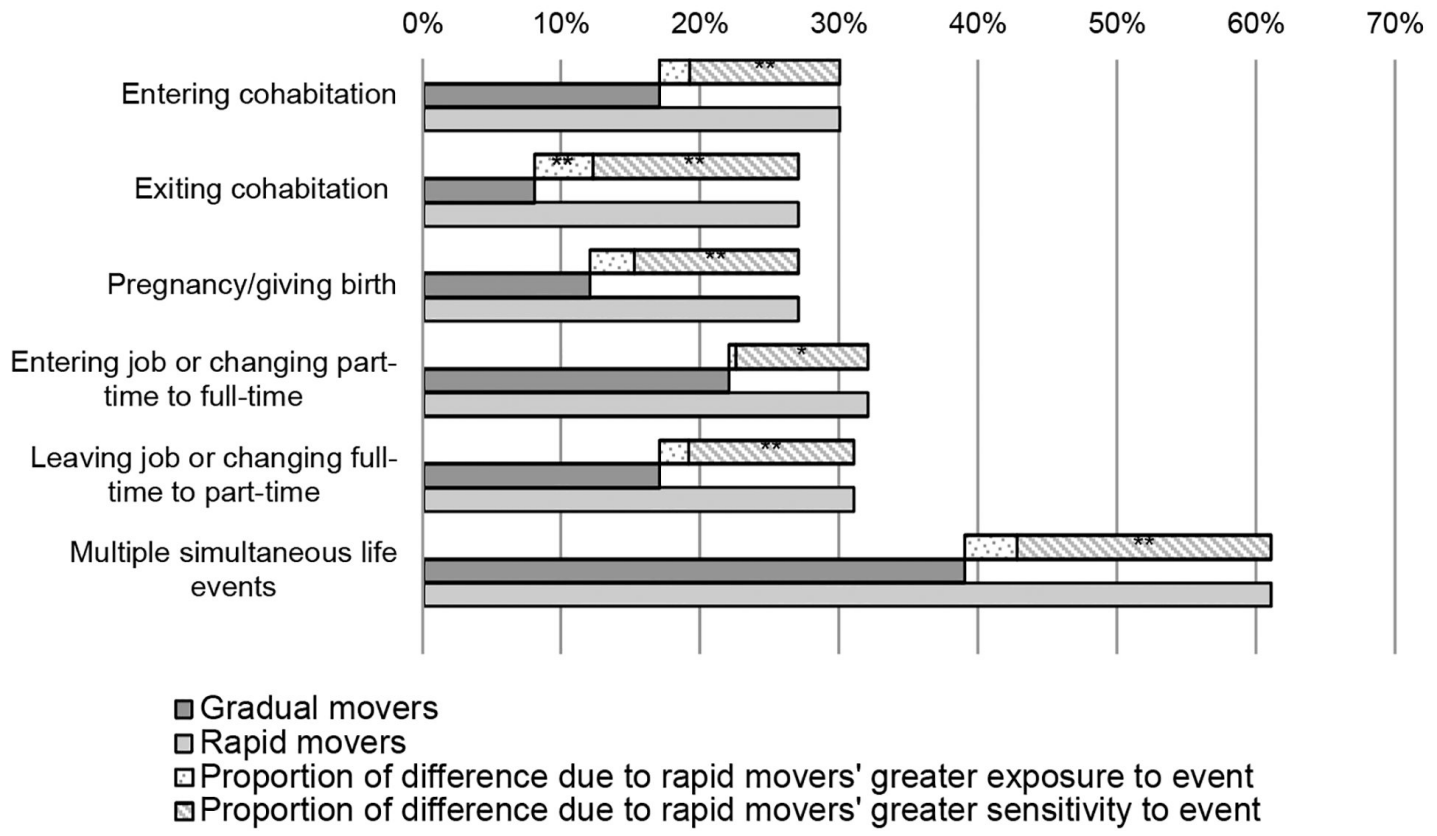
Percent of respondents who ever experienced move type and decomposition of difference across residential trajectory groups † p < .10. * p < .05. ** p < .01.

Figure 4 presents the results for a decomposition of differences in move types between gradual and rapid movers into differences in exposure to life events and differences in sensitivity to moving in the face of life events (see Table A-2 in the Appendix for results in tabular form). For any given life event, the solid, dark gray bar represents the percent of gradual movers ever experiencing the move type (i.e., moving in conjunction with the life event); the solid, light gray bar represents the percent of rapid movers ever experiencing the move type; and the sum of the shaded bars represents the difference. The dotted bar represents the proportion of the difference due to rapid movers’ greater exposure to the life event. The striped bar represents the proportion of the difference due to rapid movers’ greater sensitivity to the life event.

As the decomposition clearly demonstrates, rapid movers’ greater mobility is mostly a function of their greater sensitivity to moving in conjunction with other life events. (This pattern also holds for academic changes, although results are not shown because the overall differences in experiencing move type were only 0%–5% and therefore decomposition results were not statistically significant.) Only in the one case where the difference in exposure was significant in Figure 3 – exiting cohabitation – does the proportion of the difference due to exposure become significant.

### 4.3 Supplemental analyses

These trajectories may represent either boomeranging (moving in and out of the family home) or living with family members who are themselves housing unstable (i.e., the respondent is a member of a housing unstable household rather than the head of her own housing unstable household). RDSL did not measure household structure at every weekly interview, but rather at two points: baseline and an optional supplement one year into the study. I constructed a summary variable counting the number of moves to a known family residence. Some 15% of gradual movers and one-fifth (21%) of rapid movers moved once to a family residence. Twelve respondents in the entire sample moved twice to a family residence, eight of whom experienced four or move moves. Therefore, the vast majority of high mobility is not a function of young adults boomeranging back to stable family residences. While some respondents may be members of households that are housing unstable, this also cannot account for most residential mobility.

## 5. Discussion

This study sought to determine whether residential mobility plays a role in the reproduction of socioeconomic inequality during the transition to adulthood based on the presence of socioeconomically stratified selection and absence of moderation by differential experiences of life events. Using two and a half years of monthly mobility data for a population-representative sample of young women in a county in Michigan, I found that movers are young women who experienced poverty as children and have access to limited financial resources as young adults. Rapid movers have even lower SES than gradual movers, but the effect of SES for rapid movers is entirely mediated by relationship status. In other words, rapid movers are having accelerated transitions to adulthood that they cannot financially support. Second, I found that rapid movers and gradual movers mostly experience the same educational, employment, and romantic changes. Rapid movers do not move more frequently because they experience more life events that prompt moves. Rather, rapid movers are more sensitive to moving in the face of life changes.

These findings speak to an underlying assumption in the home-leaving literature that early home-leaving is disadvantageous – unless the destination is a college dormitory – because young adults leave home before they are prepared to support independent households. Previous research has found that early home-leaving is predicted by poverty, not growing up with two biological parents, and early family formation (Aquilino 1991; De Marco and Berzin 2008; Sandberg-Thoma, Snyder, and Jang 2015; Tang 1997). However, very little research is available on the actual aftermath or consequences of early home-leaving (for an exception, see White and Lacy 1997). The results of this study are consistent with previous findings in the home-leaving literature and begin to confirm the disadvantages associated with early home-leaving. For rapid movers, who overwhelmingly leave the family home before age 18 or 19, early home-leaving is part of an accelerated and underfunded transition to adulthood. Rapid movers then experience greater residential mobility, which is not compensated for by accompanying upwardly mobile life events.

These results are much more consistent with a housing instability perspective on high residential mobility than a socioeconomically upwardly mobile college student trajectory. Low-SES individuals do not rely on their parents for housing and often have already entered serious relationships. With greater financial responsibilities and fewer resources, they are less able to weather shocks (i.e., maintain stable housing when faced with relationship and employment changes), although for the most part they are no more likely to experience these life events.

The RDSL data set provides unprecedented detail on the residential trajectories of young women. However, I acknowledge some limitations. Significant findings in the decomposition analysis with regards to relationship and pregnancy changes but not academic changes could be a result of greater precision in that data. Weekly relationship and pregnancy changes are linked to monthly moves, as opposed to quarterly academic changes. However, this is unlikely for two reasons. First, most academic changes occur at regular, infrequent intervals (i.e., at the beginning or end of academic terms). Therefore, weekly detail is unnecessary. Furthermore, unlike exiting or entering cohabitation, the change in academic status does not necessarily occur simultaneously with the move. Moving may be anticipatory (e.g., before the school year begins) or reactionary (e.g., after graduation). Second, employment changes are measured just as infrequently but yielded significant results, suggesting that the precision of the education data did not interfere with the significance of findings.

Although the data collection site was chosen to be representative of young women in the United States in many ways, there are very few Latinas in the sample. Available statistics suggest that Latin Americans may be less likely to be homeless and more likely to be housing unstable (Conroy and Heer 2003), suggesting that they have different housing options and constraints compared to non-Hispanics. The findings from this analysis cannot be generalized to Latinas.

As demographers begin to uncover the ways in which housing can function as a mechanism for the reproduction of poverty in adulthood (Desmond 2012; Desmond and Shollenberger 2015), researchers should further explore the role of residential trajectories in contributing to stratified transition to adulthood experiences. More data collection is needed at the national level to uncover trends in residential mobility among Latinas and young men. Young men likely experience different residential mobility patterns than young women due to gendered family formation (Meier and Allen 2008; Mollborn 2007), home-leaving (Avery, Goldscheider, and Speare 1992; Buck and Scott 1993), and rental processes (Desmond 2012) that lead to gendered experiences of housing instability and homelessness (Desmond 2012; US Department of Housing and Urban Development 2014).

Future research should also capture the entire transition to adulthood. Data collection for RDSL ended when respondents were 22. This right-censoring is most significant for the rare-mover group, most of whom remained in the family home at their last observation. Likely, many members of this group exit the family home by age 25, with some young women experiencing high residential mobility. It is unclear whether housing instability is substantively different for individuals who are able to delay home-leaving – for example, because they have different relationships with their families – or if these individuals resemble the rapid group but simply exit home later.

Researchers should also disentangle the causal relationship between residential mobility and associated life events. For rapid movers, housing instability may represent either a mechanism that can partially account for well-documented educational and occupational struggles or a significant and previously unmeasured outcome of this ‘churning’ and ‘floundering’ for low-SES young adults. Data sets with self-reported reasons for move or voluntariness of move would be particularly well suited to this analysis. Such research would help determine whether direct housing interventions or improvements in educational and employment access and security would better address the cycle of instability experienced by young adults.

## Figures and Tables

**Table 1:** Logistic regressions predicting trajectory group membership: Movers (vs. rare movers) and rapid movers (vs. gradual movers)

	Movers (vs. rare movers)					Rapid movers (vs. gradual movers)				
	1	2	3	4	5	6	7	8	9	10
**Childhood background and race**										
Childhood public assistance	0.39*	0.27†	0.15	0.14	0.11	0.44*	0.37	0.29	0.29	0.26
	(0.16)	(0.16)	(0.17)	(0.17)	(0.17)	(0.20)	(0.20)	(0.21)	(0.21)	(0.21)
Did not grow up in two-parent household	0.63**	0.54**	0.44**	0.42*	0.40*	0.17	0.10	0.04	0.03	0.05
	(0.16)	(0.16)	(0.16)	(0.16)	(0.17)	(0.20)	(0.20)	(0.21)	(0.21)	(0.21)
Mother’s education less than high school	0.22	0.17	0.03	0.04	0.02	0.07	0.04	−0.09	−0.08	−0.05
	(0.26)	(0.26)	(0.27)	(0.27)	(0.28)	(0.30)	(0.31)	(0.31)	(0.32)	(0.32)
African American	−0.29†	−0.29†	−0.33*	−0.30†	−0.12	−0.49*	−0.48*	−0.54*	−0.52*	−0.38†
	(0.16)	(0.16)	(0.17)	(0.17)	(0.18)	(0.21)	(0.21)	(0.21)	(0.22)	(0.23)
**Family support**										
Financial support from family		−0.79**	−0.74**	−0.69**	−0.55**		−0.40*	−0.33†	−0.30	−0.17
		(0.17)	(0.18)	(0.18)	(0.19)		(0.20)	(0.20)	(0.21)	(0.22)
Very or extremely close to closest parent		−0.23	−0.20	−0.21	−0.26		−0.29	−0.30	−0.31	−0.34
		(0.18)	(0.18)	(0.18)	(0.18)		(0.21)	(0.21)	(0.22)	(0.22)
**Financial resources**										
Just enough/not enough money for expenses			0.40**	0.39*	0.37*			−0.05	−0.04	−0.07
			(0.15)	(0.15)	(0.15)			(0.19)	(0.20)	(0.20)
Receiving public assistance			0.34†	0.30	0.20			0.48*	0.47*	0.27
			(0.19)	(0.20)	(0.23)			(0.22)	(0.23)	(0.26)
**Education and employment**										
Enrolled in high school				−0.61*	−0.60*				−0.19	−0.16
				(0.24)	(0.25)				(0.33)	(0.33)
Enrolled in postsecondary school				−0.24	−0.21				−0.10	−0.05
				(0.18)	(0.18)				(0.22)	(0.22)
Employed				−0.10	−0.13				0.02	0.00
				(0.15)	(0.15)				(0.20)	(0.20)
**Family formation**										
Married, engaged, or cohabiting					1.22**					0.64**
					(0.24)					(0.23)
Has experienced live birth					−0.35					0.13
					(0.28)					(0.30)
**Constant**	−0.04	0.83**	0.60*	0.85**	0.64*	−0.49**	0.06	0.00	0.05	−0.20
	(0.10)	(0.23)	(0.24)	(0.29)	(0.30)	(0.15)	(0.27)	(0.29)	(0.35)	(0.36)
**Chi**^**2**^	32	57	68	75	105	11	16	21	22	30
**P**	0	0	0	0	0	0.03	0.01	0.01	0.03	0.01
**Ll**	−575	−563	−557	−554	−539	−334	−331	−329	−329	−324
**N**	868	868	868	868	868	501	501	501	501	501

*Notes*: Standard errors are shown in parentheses.

† p < .10,

* p < .05,

** p < .01.

## Appendix

**Table A-1:** Frequency of move types and simultaneity with other move types (n = 1,038 moves)

Move type	Frequency	Percent	Single event coinciding with move	Multiple events coinciding with move
**Relationship and pregnancy changes**				
Entering cohabitation	139	13%	27%	73%
Exiting cohabitation	91	9%	25%	75%
Pregnancy/giving birth	135	13%	0%	100%
**Job changes**^a^				
Entering job or changing part-time to full-time	196	19%	31%	69%
Leaving job or changing full-time to part-time	165	16%	23%	77%
**Academic changes**^a^				
Starting school	82	8%	43%	57%
Summer break	265	26%	49%	51%
Transferring to better school	61	6%	43%	57%
Transferring to worse school	35	3%	49%	51%
Graduating school	84	8%	38%	62%
Quitting school	87	8%	30%	70%
Multiple simultaneous life events	613	59%		
None of the above	142	14%		
**Total**	1,038			

Notes:

a Life events in this category are mutually exclusive.

**Table A-2:** Decomposition of difference in move quality ever experienced by respondents in gradual (n = 298) vs. rapid (n = 205) groups, by exposure and vulnerability to life events

	Percent of respondents ever experiencing life event during study (exposure)			Percent of respondents who move given exposure to life event (vulnerability)			Percent of respondents ever experiencing move in conjunction with event (exposure*vulnerability)			Percent of difference in column 9 due to:	
	1	2	3	4	5	6	7	8	9	10	11
Life events during study	Gradual	Rapid	Difference between groups	Gradual	Rapid	Difference between groups	Gradual	Rapid	Difference between groups	Exposure	Vulnerability
**Relationship and pregnancy changes**											
Entering cohabitation	53	58	5	32	51	19	17	30	13	17	83**
Exiting cohabitation	42	55	13	18	50	31	8	27	19	22**	78**
Pregnancy/giving birth	31	36	6	39	73	35	12	27	15	21	79**
**Job changes**											
Entering job or changing part-time to full-time	50	51	1	45	62	17	22	32	9	5	95*
Leaving job or changing full-time to part-time	44	48	4	38	65	27	17	31	15	15	85**
Multiple simultaneous life events	77	83	6	50	73	23	39	61	22	17	83**

*Notes*:

† p < .10,

* p < .05,

** p < .01.
